# The influence of the China GLIM standards on the diagnosis of malnutrition in patients with hematopoietic stem cell transplant

**DOI:** 10.3389/fnut.2022.1077442

**Published:** 2023-01-18

**Authors:** Feng Guo, Liu Min, Li Chengyuan, Liu Hong, Wang Meng, Tang Chenyi, Wu Jinru, Wu Wei, Liu Hua

**Affiliations:** ^1^Department of Nutrition, The Third Xiangya Hospital, Central South University, Changsha, China; ^2^Department of Hematology, The Third Xiangya Hospital, Central South University, Changsha, China; ^3^Second Department of Gastroenterology and Urology Medicine, Hunan Cancer Hospital, Changsha, China; ^4^Department of Central Sterile Supply, The Third Xiangya Hospital, Central South University, Changsha, China

**Keywords:** muscle mass, HSCT, malnutrition, GLIM-China, PG-SGA

## Abstract

**Background:**

The muscle-related indicator is removed from Global Leadership Initiative on Malnutrition (GLIM) criteria implemented in China for many reasons. Patients with hematopoietic stem cell transplants are at nutrition risk and can enter into the second step of GLIM; thus, they are suitable for learning the diagnosing malnutrition significance between primary GLIM and GLIM-China criteria. This article aims to explore the role of muscle mass in the diagnostic criteria of malnutrition and the effects of GLIM-China for diagnosing malnutrition.

**Methods:**

A total of 98 inpatients with hematopoietic stem cell transplants (HSCT) were recruited. Nutrition risk was assessed by using the Nutritional Risk Screening 2002 (NRS-2002). Appendicular skeletal muscle mass (ASMI) and fat-free mass index (FFMI) were determined using the bioelectrical impedance analysis (BIA) method. Malnutrition is defined by GLIM-China, GLIM, and PG-SGA. We use erythrocyte sedimentation rate (ESR) and C-reactive protein (CRP) to assess inflammation in GLIM and GLIM-China. The correlation or consistency among ASMI, FFMI, ESR, CRP, GLIM-China, GLIM, and PG-SGA was evaluated, respectively.

**Results:**

One hundred percent instead of the patients had nutritional risk. The magnitude of malnutrition using PG-SGA, GLIM, and GLIM-China was 75.5, 80.6, and 64.3%, respectively. GLIM-China and PG-SGA showed the same performance (*p* = 0.052 vs. 1.00) and agreement (kappa = 0.404 vs. 0.433, *p* < 0.0001) with the FFMI. Consistency was noted between ASMI and PG-SGA in the assessment of malnutrition (*p* = 0.664) with a good agreement (kappa = 0.562, *p* = 0.084). ASMI and FFMI could determine muscle mass reduction, which could not be determined by BMI, albumin (ALB), and pre-albumin (pre-ALB); 34% of GLIM-China (–) patients were with low ASMI, and 40% with low FFMI; 30.0% of patients with PG-SGA (<4) still have low ASMI, and 38.2% have low FFMI.

**Conclusion:**

If only the PG-SGA scale is used as a diagnostic criterion for evaluating malnutrition, a large proportion of patients with reduced muscle mass will be missed, but more patients with muscle loss will be missed *via* GLIM-China. Muscle-related indicators will help diagnose malnutrition.

## 1. Background

Malnutrition assessment is quite important for patients with potential nutritional risks. In 2018, muscle mass reduction was included in the Global Leadership Initiative on Malnutrition (GLIM) criteria as a diagnostic criterion. A two-step approach for the malnutrition diagnosis was selected: first, screening to identify nutrition risk status by the use of certain validated screening tools such as Nutrition Risk Screening 2002 (NRS-2002), which is based on evidence-based medicine, and second, evaluating to diagnose and alleviating the severity of malnutrition (1).

Unfortunately, it is difficult to routinely measure muscle mass due to the limitations of measurement methods in clinical work, and it is also controversial to confirm the cutoff value of muscle mass loss in the Chinese people due to the lack of evidence (2). Many researchers have tried to use the calf circumference or fat-free mass index (FFMI) tested by bioelectrical impedance analysis (BIA) as a muscle mass indicator (3), which has not yet been widely recognized. In addition, it has been suggested that muscle mass-related indicators should be removed from the phenotypic criteria by the Chinese Nutrition Screening–Undernutrition–Support–Outcome–Cost/effective (NUSOC) Group. Thus, we will refer to it as the GLIM-China criteria in the following text (2, 4, 5). Herein, we explore whether there is a difference between GLIM-China and primary GLIM in diagnosing malnutrition. All patients with hematopoietic stem cell transplant (HSCT) could enter step 2 of GLIM if they have an NRS-2002 score of 3 at least in the peri-transplant period, who are considered the target population of this study.

Before the GLIM, there were many classic criteria for diagnosing malnutrition. ESPEN recommended a body mass index (BMI) <18.5 kg/m^2^ to define malnutrition, or the combined finding of unintentional weight loss (mandatory) (weight loss >10% indefinite of time, or >5% over the last 3 months) and either a reduced BMI or a low FFMI (6). While PG-SGA is the most widely used malnutrition assessment tool for patients with cancer (7, 8), no objective and accurate data related to muscle mass are included (9).

This study aims to objectively compare the differences among muscle indicators and GLIM-China, GLIM, PG-SGA, BMI, and albumin (ALB) in the diagnosis of malnutrition, illustrating that muscle mass measurement may help diagnose malnourished patients with HSCT who are neglected by malnutrition assessment tools.

## 2. Materials and methods

### 2.1. Subjects

A total of 98 inpatients with HSCT in the hematology department were recruited from 2019 to 2020. Inclusion criteria were as follows: age ≥10 and <60 y and patients who met the hematopoietic stem cell transplantation criteria according to the evaluation of doctors in the hematology department. Exclusion criteria were as follows: pregnant and lactating patients; patients with severe infection or severe heart, liver, and kidney dysfunction; and patients with HSCT who did not agree to participate in this study.

### 2.2. Methods

Enrolled patients underwent blood sampling, body composition test, nutrition risk screening, and malnutrition diagnosis under fasting conditions under the guidance of a nutritionist. The methods are given in detail in the following paragraphs.

#### 2.2.1. Blood collection and analysis protocol

Fasting blood was collected for the measurement of albumin (ALB), ESR, and CRP. All samples were analyzed using the same reagent lot. CRP was determined by immunoturbidimetry (Beckman Image 800), and ESR was tested using the microcapillary method (ALIFAX TEST1). ALB and pre-ALB were tested by using the bromocresol green method (Hitachi, Japan).

#### 2.2.2. Body composition analysis

The body composition of recruited patients was measured by using the BIA method. The appendicular skeletal muscle mass (ASMI), fat-free mass index (FFMI), etc. were determined using the Biospace Inbody S10 composition analyzer (Biospace Co., Ltd., Seoul, Korea). ASMI measurements ≤7 kg/m^2^ for men or ≤5.7 kg/m^2^ for women were defined as low ASMI. An FFMI <17 kg/m^2^ for men or <15 kg/m^2^ for women was defined as low FFMI (10). Height and body weight were measured without shoes and under fasting, and then the body mass index (BMI) was calculated. A BMI of <18.5 kg/m^2^ was defined as a low BMI.

#### 2.2.3. Nutrition risk screening and malnutrition diagnosis

The NRS-2002 was used for the screening of nutrition risk, and an NRS-2002 score of ≥3 was suggestive of nutrition risk [NRS-2002 (+)]. Nutritional assessment was carried out using the PG-SGA scale, and a PG-SGA score of ≥4 was defined as malnutrition (9, 11).

The GLIM criteria, a two-step model for malnutrition diagnosis, containing screening and assessment, were used in our study. The primary GLIM criteria contain phenotypic (three components) and etiologic (two components) parts (12). Fulfilling at least one component in each part is necessary to diagnose malnutrition. In the phenotypic criteria, a weight loss of >5% within the past 6 months was considered positive. In Chinese patients, a BMI of <18.5 kg/m^2^ was considered low BMI if a patient was aged <70 years. Muscle mass reduction is excluded from the GLIM criteria mentioned earlier according to GLIM-China (2, 4, 5, 13).

#### 2.2.4. Statistical analysis

Statistical analysis was carried out by using SPSS version 26, and data were subjected to normal distribution analysis. Data with a normal distribution (weight, BMI, FFMI, ASMI, phase angle, BFP, BCM, and BMR) are expressed as mean ± standard deviation ($\bar{\text{x}}$ ± s) and were compared using the *t*-test. Data with a non-normal distribution (height, age, ALB, pre-ALB, VFA, CRP, ESR, NRS-2002, PG-SGA, and GLIM-China) are expressed as M+QR and were compared using the Wilcoxon rank sum test. McNemer and consistency tests were also used to examine the consistency of muscle mass indicators (ASMI and FFMI) using malnutrition diagnostic tools (GLIM-China, PG-SGA, and BMI) and biological markers (ALB and pre-ALB), respectively. The correlation among FFMI, ASMI, PG-SGA, GLIM-China, ASMI, FFMI, and CRP in the identification of malnutrition criteria of ESPEN 2015 was evaluated by using Spearman rank correlations; the relationship among ESR, CRP, PG-SGA, and GLIM-China was assessed by using logistic regression. A *P*-value of < 0.05 was considered statistically significant.

## 3. Results

### 3.1. Baseline characteristics

According to the NRS-2002, all patients with HSCT were at nutrition risk. According to PG-SGA, patients with scores of 2–3 were 24.5%, and those with scores ≥4 were 75.5%. The magnitude of malnutrition using PG-SGA, GLIM, and GLIM-China was 75.5%, 80.6%, and 64.3%, respectively (Table 1). In total, 58.16% of patients had low ASMI, 79.6% of patients had a low FFMI, 10.2% of patients had a BMI <18.5 kg/m^2^, 16.3% of patients had ALB levels lower than 35 g/L, and 35.5% of patients had pre-ALB levels lower than 200 mg/L.

**Table 1:** Nutritional status and human body composition of patients with peri-HSCT.

**Parameters**	**$\bar{\text{x}}$+*s/M+QR***
Height (m)	1.62 ± 7.82
Weight (kg)	53.40 ± 13.95
BMI (kg/m^2^)	21.35 ± 5.00
Age	39.14 ± 14.97
**Gender**	
Male	54 (55.1%)
**Methods**	
Allo-HSCT	66 (67.3%)
Autotransplantation	32 (32.7%)
**Disease**	
AML	34 (34.7%)
ALL	14 (14.3%)
MM	30 (30.6%)
Others (NHL, CML etc.)	20 (20.4%)
**NRS-2002 score**	
3	43 (43.9%)
4	12 (12.2%)
5	7 (7.1%)
6	36 (36.8%)
**PG-SGA score**	
2–3	24 (24.5%)
4–8	24 (24.5%)
≥9	50 (51.0%)
**GLIM**	
Positive	79 (80.6%)
Negative	19 (19.4%)
**GLIM-China**	
Positive	63 (64.3%)
Negative	35 (35.7%)
**Body composition**	
ASMI (kg/m^2^)	6.23 ± 1.26
FFMI (kg/m^2^)	15.36 ± 2.35
BFM (kg)	13.3 ± 6.02
BFP (%)	23.9 ± 8.71
BCM (kg)	26.10 ± 5.69
VFA (cm^2^)	63.20 ± 33.00
BMR (kcal)	1,248.3 ± 180.0
Body phase angle	4.55 ± 0.876
**Biochemical values**	
ALB (g/L)	16.96 ± 33.40
pre-ALB (mg/L)	215.40 ± 77.00
CRP (mg/L)	4.12 ± 10.76
ESR (mm/h)	31.50 ± 31.50

ASMI, appendicular skeletal muscle mass index; FFMI, fat-free mass index; BFM, body fat mass; BFP, body fat percent; VFA, visceral fat area; BCM, body cell mass; BMR, basal metabolic rate; NHL, non-Hodgkin's lymphoma; CML, chronic myeloid leukemia; CRP, C-reactive protein; ESR, erythrocyte sedimentation rate; ALB, albumin.

### 3.2. Body composition and biochemical indexes in patients with different characteristics

Appendicular skeletal muscle mass and FFMI in male patients were significantly higher than those in female patients (*p* < 0.0001). ASMI and FFMI were similar among patients treated with allo-HSCT and auto-transplantation and were not significantly different between groups with acute myeloid leukemia (AML), acute lymphocytic leukemia (ALL), multiple myeloma (MM), and other diseases (Table 2).

**Table 2:** Human body composition in patients with different characteristics.

**Variables**	**ASMI (kg/m^2^)**	**FFMI (kg/m^2^)**
**Gender**		
M	6.89 ± 1.15	16.16 ± 2.40
F	5.42 ± 0.87^****^	14.23 ± 1.69^****^
**Methods**		
Allo-HSCT	6.14 ± 1.04	15.28 ± 1.81
Auto-transplantation	6.63 ± 1.51	16.14 ± 2.79
**Disease**		
AML	6.45 ± 0.25	15.45 ± 0.43
ALL	5.84 ± 0.28	14.18 ± 0.52
MM	6.14 ± 0.20	15.27 ± 0.34
Others (CML, NHL etc.)	6.28 ± 0.30	15.47 ± 0.50

**** *p* < 0.0001, female vs. male patients.

Female patients had lower pre-albumin (*p* < 0.0001) and albumin concentrations than male patients (*p* = 0.0054), but there was no significant difference in CRP and ESR between the male and female patients. The pre-albumin of patients with autologous stem cell transplant was higher than that of patients with allogeneic hematopoietic stem cell transplantation (*p* = 0.0362). No significant difference was found in ALB, CRP, and ESR levels between the two transplantation methods. Patients with AML had higher pre-albumin concentrations than patients with MM (*p* = 0.0088), and patients with AML had higher albumin concentrations than patients with MM (*p* = 0.0244) (Table 3).

**Table 3:** Serum biomarkers in patients with different characteristics.

**Variables**	**ALB (g/L)**	**pre-ALB (mg/L)**	**CRP (mg/L)**	**ESR (mm/h)**
**Gender**				
M	39.50 ± 4.12	224.95 ± 67.84	7.00 ± 9.16	33.09 ± 26.76
F	37.04 ± 4.41^**^	191.29 ± 45.72^****^	12.82 ± 19.23	36.02 ± 22.12
**Methods**				
Allo-HSCT	37.56 ± 3.39	202.03 ± 39.81	9.39 ± 17.62	35.78 ± 22.34
Autotransplantation	40.86 ± 4.26	228.72 ± 78.14	5.74 ± 13.62	21.47 ± 18.04
**Disease**				
AML	40.14 ± 0.87	237.2 ± 10.10	10.03 ± 2.67	32.29 ± 4.16
ALL	36.09 ± 1.15	245.5 ± 31.90	7.29 ± 2.34	28.08 ± 6.59
MM	37.61 ± 0.63	202.3 ± 7.65	9.19 ± 3.31	34.97 ± 4.12
Others (CML, NHL etc.)	38.23 ± 0.81^a^^b^^**^	209.7 ± 12.14^c^^**^	11.81 ± 2.97	41.20 ± 6.52

** *p* = 0.0054 female vs. male patients;

**** *p* < 0.0001, female vs. male patients.

a *p* = 0.0118, AML vs. ALL.

b *p* = 0.0244, AML vs. MM.

c *p* = 0.0088, AML vs. MM.

### 3.3. ASMI and FFMI were consistent in malnutrition assessment using diagnostic tools of PG-SGA and GLIM-China

According to McNemer and consistency tests, inconsistent results were noted between FFMI and BMI in the assessment of malnutrition (*p* < 0.001), and there was little agreement between the FFMI and BMI (kappa = 0.274, *p* < 0.0001). FFMI and ALB were inconsistent in the assessment of malnutrition (*p* < 0.0001), with a poor agreement (kappa = 0.12, *p* = 0.094). FFMI and pre-ALB were also inconsistent in the assessment of nutritional status (*p* < 0.001), with a similar poor agreement (kappa = 0.122, *p* = 0.168). Interestingly, the results were consistent between FFMI and GLIM-China in the assessment of malnutrition (*p* = 0.052), with a moderate agreement (kappa = 0.404, *p* < 0.0001). The positive rate determined by FFMI (66.3%) was higher than that by GLIM-China (64.3%); thus, there was a trend toward significantly different results. We also found consistent results between FFMI and PG-SGA in the assessment of malnutrition (*p* = 1.00), with a fair agreement (kappa = 0.433, *p* < 0.0001). The positive rate determined by FFMI (66.3%) was slightly higher than that by PG-SGA (65.3%), but no significant difference was found (Table 4).

**Table 4:** Consistence of ASMI and FFMI with nutritional scales/parameters in the nutritional assessment.

**BIA**	**ALB**		**Pre-ALB (93, missing** = **5)**		**BMI**		**PG-SGA**		**GLIM-China**		**Total**
	+	**–**	+	**–**	<**18.5**	≥**18.5**	<**4**	≥**4**	+	**–**	
Low ASMI	20 (83.3)	37 (50.0)	23 (69.7)	30 (50.0)	24 (92.3)	33 (45.8)	48 (70.6)	9 (30.0)	45 (71.4)	12 (34.3)	57 (58.2)
Normal ASMI	4 (16.7)	37 (50.0)	10 (30.0)	30 (50.0)	2 (7.7)	39 (54.2)	20 (29.4)	21 (70.0)	18 (28.6)	23 (65.7)	41 (41.8)
Low FFMI	20 (80.0)	45 (61.6)	24 (72.7)	36 (60.0)	25 (96.2)	40 (55.6)	52 (81.3)	13 (38.2)	50 (79.4)	19 (48.7)	65 (66.3)
Normal FFMI	5 (20.09)	28 (38.4)	9 (27.3)	24 (60)	1 (3.8)	32 (44.4)	12 (18.8)	21 (61.8)	13 (20.6)	20 (51.3)	33 (33.7)
Total	24 (24.5)	73 (75.5)	60 (64.5)	33 (35.5)	26 (26.5)	72 (73.5)	74 (74.7)	25 (25.3)	63 (64.3)	35 (35.7)	98 (100)

Inconsistency was noted between ASMI and BMI in the assessment of malnutrition (*p* < 0.001), and there was low agreement (kappa = 0.337, *p* < 0.001), comparing ASMI instead with ALB and pre-ALB, we also found no consistency between ASMI and ALB (*p* < 0.001; kappa = 0.228, *p* = 0.004), and ASMI and pre-ALB (*p* = 0.002; kappa = 0.173, *p* = 0.066). Significant consistency was noted between ASMI and PG-SGA in the assessment of malnutrition (*p* = 0.664), and there was good agreement between ASMI and PG-SGA (kappa = 0.562, *p* < 0.0001). The positive rate determined by ASMI (58.2%) was slightly lower than that by PG-SGA (60.6%), but these results were not significantly different. Consistency was found between ASMI and GLIM-China in the assessment of malnutrition (*p* = 0.362), with a poor agreement (kappa = 0.358, *p* < 0.001), and the positive rate determined by ASMI (58.2%) was lower than that by GLIM-China (64.3%, *p* < 0.001) (Table 4).

### 3.4. Correlations of body composition with PG-SGA and GLIM-China

Further correlation analyses revealed a moderate negative relationship between FFMI and PG-SGA (*r*_s_ = −0.513, *p* < 0.0001). This negative relationship was noted in both male (*r*_s_ = −0.204, *p* = 0.142) and female patients (*r*_s_ = −0.4956, *p* = 0.001). A negative relationship was noted between ASMI and PG-SGA (*r*_s_ = −0.480, *p* < 0.0001), and this negative relationship was present in both male (*r*_s_ = −0.247, *p* = 0.075) and female patients (*r*_*s*_ = −0.515, *p* < 0.0001). There was also a negative relationship between FFMI and GLIM-China (*r*_s_ = −0.480 *p* < 0.0001) and present in male patients (*r*_s_ = −0.115 *p* = 0.411) but not in female patients (*r*_s_ = −0.519, *p* < 0.0001). A negative relationship between ASMI and GLIM-China (*r*_s_ = −0.372, *p* < 0.0001) and present in male patients (*r*_s_ = −0.139, *p* = 0.322) but not in female patients (*r*_s_ = −0.439, *p* = 0.003).

### 3.5. Correlation between biochemical criteria (ESR and CRP) and GLIM-China, PG-SGA

We used logistic regression to evaluate the effect of ESR and CRP on the diagnosis of malnutrition in GLIM-China. The result of the logistic regression model was statistically significant [$\chi_{\left(4\right)}^{2}$ = 6.487, *p* < 0.05] as the model explained 8.9% of the variation (Nagelkerke R2) with or without malnutrition and was able to correctly classify 65.6% of the patients. The sensitivity of the model was 91.8%, the specificity was 20.0%, the positive predictive value was 65.3%, and the negative predictive value was 56.8%. Equally, the logistic regression model of ESR, CRP, and PG-SGA was statistically significant [Table 5; $\chi_{\left(4\right)}^{2}$ = 11.407, *p* = 0.003], and this model explained 16.8% of the variation (Nagelkerke R2) with or without malnutrition and was able to correctly classify 76% of patients. The sensitivity of this model was 100%, the specificity was 0.00%, the positive predictive value was 74.5%, and the negative predictive value was 0.00%.

**Table 5:** Logistic analysis between CRP/ESR and GLIM-China/PG-SGA.

	**B**.	**S.E**.	**Wald**	**df**	***p***	**Odds ratio**	**95%CI for odds ratio**	
							**Lower**	**Upper**
**GLIM-China**								
CRP	0.014	0.021	0.457	1	0.499	0.972	0.973	1.057
ESR	0.020	0.011	3.266	1	0.071	1.048	0.998	1.043
**PG-SGA**								
CRP	0.012	0.028	0.185	1	0.139	1.012	0.959	1.068
ESR	0.039	0.015	6.410	1	0.004	1.040	1.009	1.072

Taken together, ESR and CRP had higher sensitivity to malnutrition than GLIM-China and PG-SGA, but the specificity was low, and the prediction of PG-SGA for malnutrition was better. Compared with PG-SGA, the false-negative rate of ESR and CRP was lower, and the false-positive rate was similar to that of GLIM-China.

## 4. Discussion

During hematopoietic stem cell transplantation, abnormal taste, poor appetite, and impaired digestion, as well as a high magnitude of malnutrition, occurred in patients with HSCT. The prospective longitudinal cohort study by Barritta de Defranchi et al. showed that 59.7% of patients with HSCT were malnourished. In our study, we found that the malnutrition magnitude differences among the PG-SGA scale, GLIM criteria, and GLIM-China were 75.5, 80.6, and 64.3%, respectively, which was basically consistent with the study by Barritta de Defranchi et al. (14) and Brotelle et al. (15).

The definition of malnutrition is debated recently. No single existing approach has secured broad global acceptance (6, 16–20). The advantage of GLIM is that it can evaluate the nutritional status more simply and accurately by incorporating objective muscle mass data into the evaluation. However, the FFMI and ASMI cutoff values measured by using the BIA method are not based on Chinese population standards. Therefore, some researchers in China define malnutrition by using the GLIM criteria without muscle mass data, as mentioned earlier (2). However, Jingyong Xu showed that nutritional support therapy after the GLIM assessment removed muscle mass and neglected the benefits of reducing infection complications (13). In our previous research, we also found that some IBD patients with muscle mass reduction cannot be identified by commonly used nutrition assessment scales such as NRS-2002 (21). Our team has considered whether GLIM-China has an impact on malnutrition diagnosis. We also found a suitable population to confirm the role of muscle mass in malnutrition diagnosis. According to the NRS-2002 part of “Severity of disease,” patients with HSCT at least have a score of 3, indicating that patients with HSCT are all at nutrition risk, and thus can enter into the second step of GLIM; hence, they are suitable for learning the diagnosing malnutrition significance between primary GLIM and GLIM-China criteria.

Appendicular skeletal muscle mass and FFMI were recommended by ESPEN. BIA, which had good consistency with DEXA, was used to measure the ASMI and FFMI of patients with HSCT (22). We found that normal FFMI (FFMI ≧ 17 kg/m^2^ for men or >15 kg/m^2^ for women) and GLIM-China (-) diagnosed malnutrition were generally consistent with each other, possibly related to the inclusion of FFMI in the process of GLIM-China. Consistency was shown between ASMI and GLIM-China in the assessment of malnutrition but with a poor agreement. The low ASMI rate (58.2%) is lower than the GLIM-China (+) rate (64.3%). So, GLIM-China cannot be replaced by ASMI because many nutrition-related factors are included in it such as weight, food intake, inflammation, and disease.

**Figure 1:**
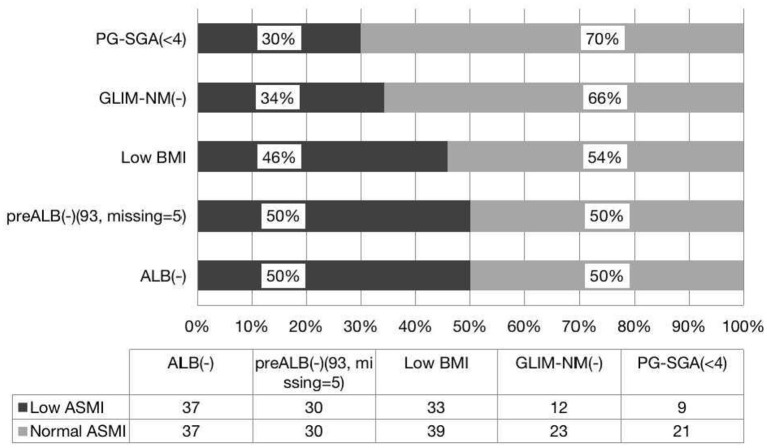
Consistence of the ASMI with the nutritional scales/parameters in the nutritional assessment of patients with HSCT.

**Figure 2:**
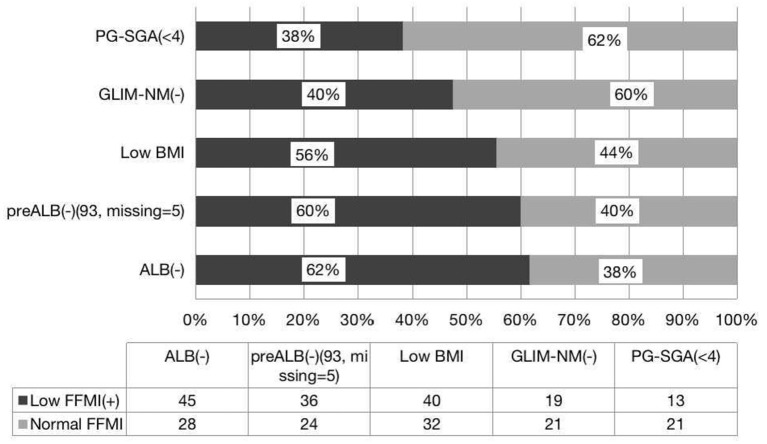
Consistence of the FFMI with the nutritional scales/parameters in the nutritional assessment of patients with HSCT.

PG-SGA is a widely used tool to detect patients with malnutrition or at risk of malnutrition (9). We found that PG-SGA and ASMI are parallel in the diagnosis of malnutrition, and there is good agreement between the two methods. Similarly, PG-SGA and FFMI are consistent in the diagnosis of malnutrition, but there is no difference between the positive rates. In this study, we found that 38.2% of patients had normal nutrition by PG-SGA but with low FFMI, and 30% of whom had low ASMI. Interestingly, 40% of patients had normal nutrition by GLIM-China with low FFMI, and 34% had low ASMI. It is clear that if only the PG-SGA scale is used as a diagnostic criterion for evaluating malnutrition, a large proportion of patients with reduced muscle mass will be missed, but a larger number of patients will be missed by GLIM-China. If the value of ASMI and FFMI is included in the GLIM criteria, patients with low FFMI or low ASMI can be diagnosed with GLIM (+), which may effectively avoid the missed diagnosis of malnutrition. Therefore, compared with GLIM-China and PG-SGA, we propose that both FFMI and ASMI can also be used to diagnose malnutrition. GLIM-China is less sensitive than PG-SGA for diagnosing malnutrition in patients with HSCT (Figures 1, 2). If the standard of FFMI and ASMI is adopted in GLIM, the positive result of GLIM will be the same as the result showed low FFMI and low ASMI. In addition, compared with GLIM, using GLIM-China may lose some patients who need nutritional therapy.

Hypoproteinemia increases bacteremia and mortality in patients with hematopoietic stem cell transplants. The albumin level in serum is affected by many factors such as inflammation, infection, liver damage, and fluid status. Therefore, albumin was no longer recommended for identifying malnutrition by the Academy of Nutrition and Dietetics (AND) and ASPEN (1, 19). We set 35 g/L as the cutoff value for this study because it has commonly been used as evidence of malnutrition in hospitalized elderly patients (23). Our data indicated that hypoproteinemia occurred in 16.3% of patients during peri-hematopoietic stem cell transplantation (24), which was less than the positive rate of PG-SGA, GLIM, and GLIM-China. Compared with albumin, serum pre-albumin is considered a more sensitive indicator of nutritional status, which has also been used as a blood marker for malnutrition. A meta-analysis revealed that pre-albumin concentrations <20 mg/dL may indicate malnutrition (25), so we chose this value as the cutoff value for the current study. Our study found that 35.5% of patients had low pre-albumin levels and 50% of those with normal pre-albumin had low ASMI, 60% of those with normal pre-albumin patients had low FFMI, 50% of those with normal albumin had low ASMI, and 61.6% of those with normal albumin had low FFMI. Thus, these results indicated that ASMI and FFMI cannot be replaced by albumin and pre-albumin.

Inflammation is listed in the GLIM as one of the indicators that may cause malnutrition. It has been suggested that the loss of muscle mass may be related to changes in skeletal muscle mitochondria, leading to ROS generation-mediated inflammation-induced skeletal muscle cell apoptosis (26, 27). CRP reflects the level of acute inflammation (28), and our samples were generally collected when patients' condition was relatively stable, such as before HSCT or 2 weeks after the infusion of stem cells. The ESR is another widely used inflammation indicator. Since the ESR does not change rapidly at the beginning of the inflammation process, and the normalization rate is slower than other acute-phase reactants, we also analyzed the ESR and muscle mass reduction. However, we found that CRP and ESR had no correlation with the decline of FFMI and ASMI. Therefore, muscle mass reduction cannot be replaced by serum inflammation indicators. Similarly, regression analysis results suggested that both CRP and ESR are lacking specificity in the diagnosis of malnutrition.

One limitation of our research is the relatively low number of enrolled patients. Due to the lack of large prospective randomized controlled study data to identify the cutoff value of muscle mass reduction for Chinese patients, we just used the sarcopenia standard of Asia (10), which may have caused some bias. More prospective studies with larger sample sizes are needed to confirm our findings.

## 5. Conclusion

A high nutrition risk rate (100%) and malnutrition prevalence rate are common among patients with HSCT; FFMI and ASMI are helpful for finding malnourished patients with HSCT who are missed by the PG-SGA scale and GLIM-China. If only the PG-SGA scale is used as a diagnostic criterion for evaluating malnutrition, a large proportion of patients with reduced muscle mass will be missed, but more patients with muscle loss will be missed *via* GLIM-China.

## Data availability statement

The original contributions presented in the study are included in the article/supplementary material, further inquiries can be directed to the corresponding authors.

## Ethics statement

The studies involving human participants were reviewed and approved by NCT04591340. Written informed consent to participate in this study was provided by the participants' legal guardian/next of kin.

## Author contributions

FG and LM designed this study. FG and LC performed the research, analyzed data, and wrote the manuscript. FG, LC, LM, LHo, TC, WM, LHu, WJ, and WW collected the study data. All authors read and approved the final version of this manuscript.
